# Effects of Pain Neuroscience Education and Physiotherapy on Chronic Low Back Pain, Fear of Movement and Functional Status: A Randomised Pilot Study

**DOI:** 10.3390/jcm13072081

**Published:** 2024-04-03

**Authors:** Eglė Lendraitienė, Barbora Styraitė, Rasa Šakalienė, Gabija Misytė, Indre Bileviciute-Ljungar

**Affiliations:** 1 Department of Rehabilitation, Lithuanian University of Health Sciences, LT-44307 Kaunas, Lithuania; egle.lendraitiene@lsmu.lt (E.L.); barbora.st1998@gmail.com (B.S.); gabija.misyte@gmail.com (G.M.); 2 Department of Clinical Sciences, Karolinska Institutet at Danderyd University Hospital, 18288 Stockholm, Sweden; 3 Multidisciplinary Pain Clinic, Capio St. Göran Hospital, 11219 Stockholm, Sweden

**Keywords:** kinesiophobia, physiotherapy, non-specific low back pain, pain neuroscience education, disability

## Abstract

**Background:** Chronic non-specific low back pain is a non-harmous condition often found in the general population. It is one of the most significant disabilities and needs different treatment modalities. This study investigates the effects of pain neuroscience education and physiotherapy on pain intensity, fear of movement and functional status in a Lithuanian cohort with non-specific low back pain. **Methods:** The study was performed at the primary health care unit in Kaunas, Lithuania. The key inclusion criterion was persistent non-specific low back pain longer than three months and which affects daily life functions. Thirty participants (mean 33.47, SD 4.38 years age, 70% women) were randomised into two training groups with and without pain neuroscience education (for a total of 60 min of teaching). Physiotherapy was performed twice per week during 45 min/session for a period of 10 weeks with exercises which strengthen, stabilize, and stretch the spinal cord muscles. Outcomes included pain intensity, kinesiophobia and disability and these were measured by self-scored questionnaires (numeric rating scale, Tampa scale for kinesiophobia-11, Oswestry disability index and the Roland–Morris questionnaire, respectively). **Results:** The results indicate that both groups improved in the measured outcomes, with the only difference between them being a better improvement in kinesiophobia in the group receiving physiotherapy and pain neuroscience education. **Conclusions:** The results of this study confirm that a relatively short intervention of pain neuroscience education enhances the effects of physiotherapy and should be implemented in clinical practice.

## 1. Introduction

According to scientific data, about 90 percent people have encountered lower back pain at least once in their life [1]. Low back pain (LBP) is described in the literature as pain, muscle tension, or stiffness felt between the lower costal arch and the lower ischial folds, with or without spreading to the lower extremities [2]. LBP can be acute, subacute and chronic. Chronic low back pain (CLBP) has several categories. One of these is non-specific low back pain (NLBP), which is characterized by the fact that no specific pathological anatomical causes of the pain are determined [1]. According to statistics, CLBP occurs in 90% of cases [3]. It is scientifically proven that chronic non-specific low back pain is the result of anatomical, structural and biomechanical changes in the spine [4]. The treatment of chronic non-specific lower back pain is complex. Based on the fact that the origin of pain is often related to the musculoskeletal system, one of the most effective methods of reduction is physiotherapy [5,6]. During active physiotherapy, specific exercises are applied, including stabilization exercises [7,8,9], muscle strength training exercises [10,11], stretching exercises [12,13], and aerobic exercises [14].

Another, increasingly more frequently applied method is pain recognition education [15]. Research has shown that people who experience pain are interested in pain, especially in how pain works. Educating people about the neuroscience related to pain has been observed to have positive therapeutic effects, with the term pain neuroscience education (PNE), based on pain cognition training (PCT) [16], has been introduced. PCT is a cognitive intervention, a strategy that aims to change patients’ perception of pain. Its goal, first of all, is to enlighten patients about the origins and causes of pain itself and, using various stories and metaphors, to help patients re-understand their pain experience. This type of training is aimed at increasing the pain threshold during exercises, reducing kinesiophobia, and developing pain-related brain centres.

The focus of PNE is on reconceptualizing pain by educating patients about the neurobiological and neurophysiological processes involved in their pain experience rather than focusing solely on tissue pathology [17]. Therefore, PNE emphasizes that chronic pain is caused by neuroplasticity in the brain, which leads to increased sensitivity of the central nervous system, otherwise known as central sensitization. Specifically, PNE includes education about patient perceptions of the cause of pain, the onset of pain, and how to reduce pain [18]. When we educate patients about their pain, our aim is to change the context of how they perceive it by providing this new information. Studies have shown that manipulating the information and context surrounding stimulation can modulate pain [19]. Based on a large number of studies, the greatest therapeutic benefit is achieved when PNE is combined with physiotherapy. For example, a recent study by Meise et al. has shown that physiotherapy, in combination with PNE, is superior to physiotherapy alone in terms of the reduction of the headache frequency of adult patients with migraine. Furthermore, a better understanding of the disease and its underlying pathophysiology may facilitate a reduction of fear of the next attack of pain [20]. However, the results of a literature review by Puentedura et al. show that chronic lower back pain was more effectively reduced by using manual methods only, when compared with the application of pain awareness education with manual therapy [17].

Considering the statistics, CLBP afflicts a large part of the population [1,2], which makes it worthwhile to search for, modify and incorporate new treatment methods into the treatment plan. The aim of the research is to determine whether physiotherapy, combined with pain neuroscience education is more effective than physiotherapy alone. The following measures related to NLBP were chosen as primary outcomes: pain intensity, kinesiophobia and disability.

## 2. Materials and Methods

### 2.1. Participants and Questionnaires

A randomized clinical trial was conducted in November 2022–January 2023 in a medical institution in Kaunas, Lithuania. The approval of the Bioethics Centre of the Lithuanian University of Health Sciences, no. BEC-SR(M)-100, was obtained on the 20th of December 2020. All study participants gave their written consent to participate. The sample comprised 30 people. The recruitment was undertaken by an announcement on a noticeboard or by informing new patients visiting the institution. Inclusion criteria were as follows: (1) age between 25–40 years, (2) no spine disorders or trauma, (3) persistent pain longer than 3 months, (4) not taking any prescribed or regular unprescribed medication against low back pain; and (5) undersigned consent of participation in the study. Exclusion criteria were as follows: not fulfilling inclusion criteria and not able to participate in 10 weeks of training exercises. A CONSORT 2010 flow diagram is presented as Supplemental Figure S1. Participants in group I consisted of 8 women and 7 men (n = 15) and they received only physiotherapy, which was administered twice a week for 45 min for a period of 10 weeks. This physiotherapy was in the form of a program that consisted of muscle strengthening, stabilization and stretching exercises. In group II, which consisted of 11 women and 4 men (n = 15), physiotherapy was combined with an educational one-hour program of pain neuroscience presented on slides. The subjects’ pain and all functional status tests were performed twice—before and after the intervention, i.e., after 10 weeks (Table 1).

The following methods were used during the study: a questionnaire survey, assessment of pain intensity using the numeric rating scale (NRS), assessment of fear of movement using the Tampa scale for kinesiophobia-11 (TSK-11), assessment of the impact of back pain on the patient’s functional capacity according to the Oswestry disability index, and assessment of functional disability using the Roland–Morris questionnaire (Table 2).

### 2.2. Physiotherapy Program

**Figure 1:**
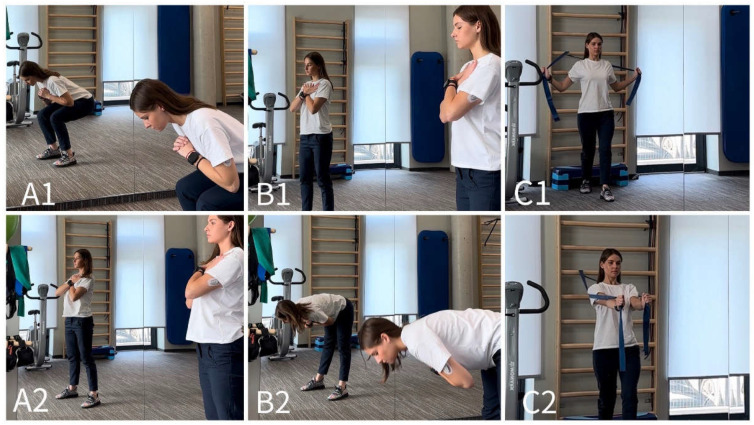
Muscle strength training exercises while standing. Squats (**A1**,**A2**), stiff-legged deadlifts (**B1**,**B2**) chest fly (**C1**,**C2**). © Gabija Misytė.

**Figure 2:**
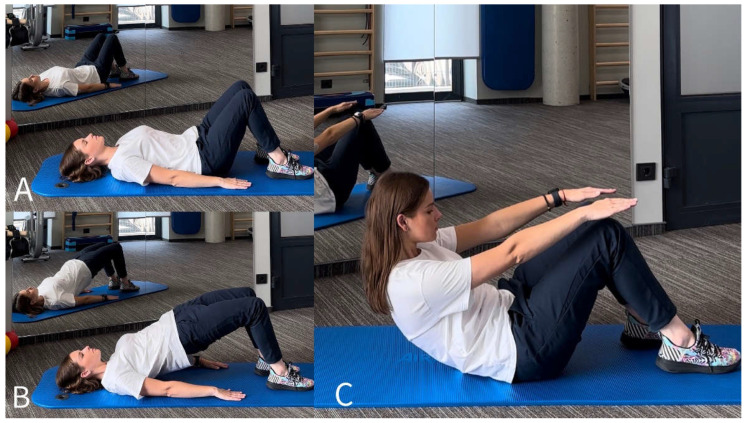
Muscle strength training exercises while lying down. Pelvic tilt (**A**), isometric pelvic bridge (**B**), sit-up (**C**). © Gabija Misytė.

The physiotherapy program consisted of muscle strengthening, spinal stabilization and stretching exercises. Physiotherapy was performed in the physiotherapy room. An educational program was also taught in the same hall using the technologies available there. The selected exercise load was individualized for each subject according to physical capabilities while maintaining the correctness of the performed exercise. Muscle strengthening training exercises were performed with body weight or elastic resistance bands for the main muscle groups (Figure 1 and Figure 2) [26,27]. The spinal stabilization exercise program, which consisted of five exercises, was based on a scientific publication prepared by Boucher et al. [28]. The adherence was 100%, meaning that all of the participants fulfilled their individual programs. However, some participants did miss a number of sessions and compensated for this during their next session, for example regarding PNE and missed exercise training. Adverse effects were not reported.

### 2.3. Pain Neuroscience Education Program

The program consisted of six topics: differences between acute and chronic pain, nociceptive processes, performance potential, peripheral and central sensitization, pain suppression processes, and plasticity of the nervous system. For the participants, the material was divided into two parts of 30 min each and was taught before the physiotherapy intervention. The program was both prepared in slides and presented by the organizer of the study.

### 2.4. Statistics

Statistical Package for Social Science (SPSS) version 29.0 was used for statistical analysis of research data. Quantitative data are presented as minimum value (min), maximum value (max) and as mean with standard deviation (m ± SD). After evaluating the sample size of the subjects in the groups and performing the Shapiro–Wilks normality test, a decision was made to use non-parametric tests. The χ² test was used to find differences in the distribution of nominal variables between population groups. The non-parametric Wilcoxon (Z) test was used to calculate within-group differences. One-way ANOVA was used to calculate between-group differences of absolute values and intra-group changes. 

The difference was considered to be statistically significant when *p* < 0.05.

## 3. Results

### 3.1. Sociodemographic Data

The average age of subjects in group I was 33.1 (±4.37) years, and the mean age of subjects in group II was 33.8 (±4.51). There was no difference in age between the groups (*p* = 0.834). The study revealed that 43.3 percent (n = 13) subjects complain of lower back pain every day, 36.7 percent (n = 11) once or several times a week, and 20 percent (n = 6) feel this type of pain less often. All 30 subjects noted that they had lower back pain for more than three months and none of them were currently diagnosed with any spinal injury or disease. Of the subjects, 96.7 percent (n = 29) stated that this pain limits their daily activities and functions, and 23.3 percent (n = 7) noted that they use medication for lower back pain. Moreover, approximately 40 percent (n = 12) of participants reported that they were in a “sitting” position during work, 16.7 percent (n = 5) pointed out they undertook physical work, 3.3 percent (n = 4) were in a “standing“ position at work, 13.3 percent (n = 4) a mixed “sitting” position with physical work, 10 percent (n = 3) a mixed “standing” position with physical work, and 6.7 percent (n = 2) a mix of “standing” and “sitting” positions. When examining demographic and other factors, no significant differences were found according to the examined groups of subjects (*p* > 0.05). Among the subjects, there were no such advanced comorbidities that would have necessitated changes in exercise or a reduction in exercise intensity. The results are presented in Table 3.

### 3.2. Assessment of the Outcomes Measures

**Figure 3:**
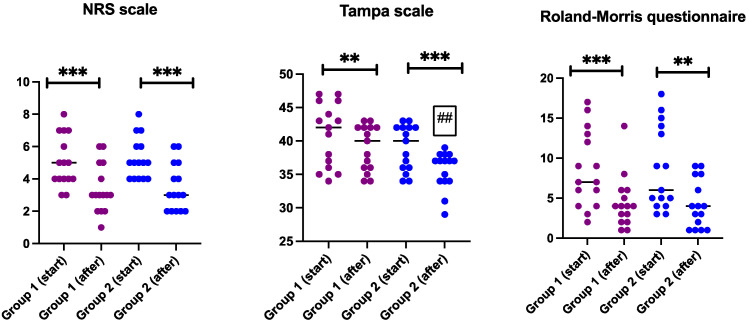
Data are presented as individual values (with line at median) of numeric rating pain scale (NRS), Tampa scale and Roland–Morris questionnaire. ** *p* < 0.01 and *** *p* < 0.001 indicates differences within the group (Wilcoxon test); ## indicates *p* < 0.01 difference between the groups (one-way ANOVA). Group 1 = physiotherapy without PNE and group 2 = physiotherapy with PNE.

Figure 3 presents individual scores of pain intensity (NRS), kinesiophobia (Tampa scale for kinesiophobia-11) and low back pain-related disability (Roland–Morris questionnaire), showing that all measurements significantly improved in both groups. The only differences found between the groups were associated with our use of the Tampa scale. Participants in the physiotherapy with PNE group scored significantly higher in terms of improvement as compared with the physiotherapy without PNE, both for absolute values (Figure 3) and for intra-group change (6.3 vs. 1.9, *p* < 0.001, results not shown).

**Figure 4:**
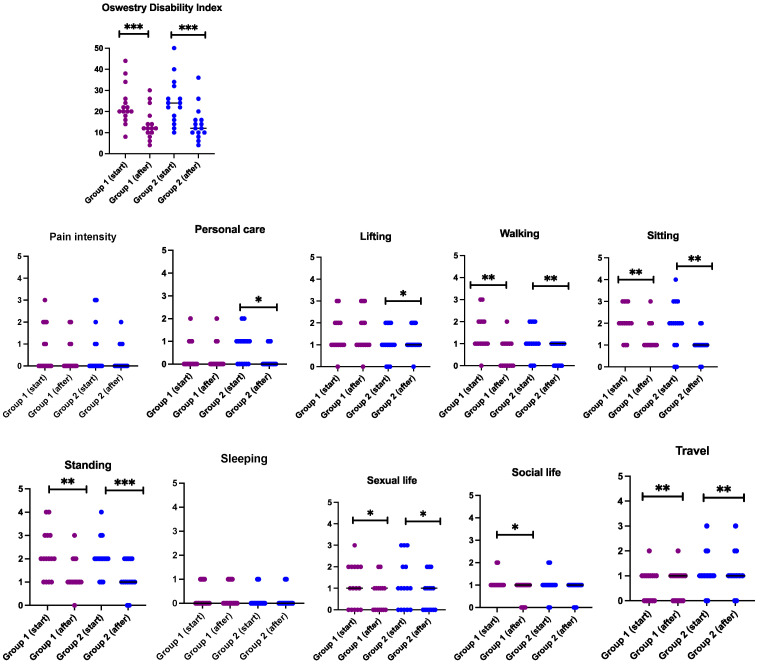
Data are presented as individual values (with line at median) of the Oswestry disability index (total score), including ten subscales. * indicates differences within the group (Wilxocon test); * *p* < 0.05; ** *p* < 0.01 and *** *p* < 0.001. Group 1 = physiotherapy without PNE and group 2 = physiotherapy with PNE.

Results of the Oswestry disability index are presented in Figure 4, showing a significantly statistical improvement in both when measuring the total score. Results of ten subscales revealed improvements in walking, sitting, standing, sexual activity and traveling in both groups, while personal care and lifting were improved in physiotherapy with PNE group only. Social life was improved in physiotherapy without PNE only. No differences were found between the groups. When analysing intra-group changes, the subscale of personal care was improved more in the physiotherapy with PNE group as compared with that of physiotherapy without PNE, at 0.5 vs. 0.07, respectively (*p* < 0.029, results not shown).

## 4. Discussion

The results of this study show that the physiotherapy program, consisting of spinal stabilization, muscle strength and stretching exercises, applied both separately and together with pain recognition training, had a positive effect on chronic non-specific LB pain reduction, but few significant differences were detected between the groups. When combining physiotherapy with pain recognition training, the fear of movement decreased more than in the group receiving only physiotherapy. After assessing the influence of back pain on the patients’ functional ability using the Oswestry disability index, positive changes were determined in both the first and the second groups. In the overall results, no statistically significant difference was detected between the groups, except for one component—self-service (intra-group difference only). We discovered that, after the applied intervention, the indicators of this component were better in the subjects of group II. Analysis of the Roland–Morris questionnaire before and after the study revealed that the functional status improved equally in both groups, the results were not significantly different.

Compared with the insights of other authors, there are both similar and completely different research results. For example, in a study by Gorji et al., an educational program with motor control exercises was found to be more effective in reducing CLBP than stabilization exercises [29]. Similar results have been presented by Malfliet et al., who stated that motor control exercises and education about pain had a greater benefit in reducing back pain than typical or traditional physiotherapy [30]. However, the effects of PNE *per se* were not evaluated in those two latter studies. Kim et al. discovered that spinal stabilization exercises, combined with pain neuroeducation, reduced pain in participants to a greater extent than physical exercises alone [31]. Although a significant number of studies have demonstrated the benefits of an educational program in reducing pain in CLB, it has been suggested that there is a lack of research examining these types of programs. The following questions arise: what aspects of the program would be most effective? What should be the focus of education [14,30,31]? Watson et al. conducted a review of 23 qualitative studies and identified several key components that make up a pain education program. According to them, one of the most important nuances is to allow the patient to tell their story and experiences in order to analyse their understanding of pain itself as a phenomenon and to individualize the training accordingly [32]. Meanwhile, Wijma et al. have stated that, before introducing the patient to education about pain, it is necessary to perform a biopsychosocial assessment of them (the patient) in order to classify it [33]. This model attempts to clearly determine the dominant pain mechanism (predominant nociceptive, neuropathic or non-neuropathic pain of central sensitization), as well as to evaluate the provocative and long-term biopsychosocial factors in patients with CLB pain [33].

Another indicator researched in the study was fear of movement. When evaluating the results of other authors’ research, similarities can be seen. In a study on CLBP, physiotherapies with and without pain recognition training were compared [34]. The physiotherapy programs were applied five times a week for three weeks, and the educational material was taught twice a week for three weeks. The results show that, in the group in which there was a combination of the two methodologies, there was a statistically significant decrease in fear of movement and an increase in muscle endurance [34]. The findings by the other authors show that pain recognition training and motility control exercises improved the functional status of patients more than stabilization exercises alone [32]. Siddall et al. conducted a systematic review of five high-quality studies and made a conclusion that the combination of physical exercises and pain recognition education is an effective tool for increasing the patient’s functional independence. Additionally, they emphasize that the physiotherapy program for patients with LBP should not be limited to one methodology and thus encourage the use of various exercises [35].

After analysing the obtained results, we can state that our hypothesis has been partially confirmed. Physiotherapy, applied both alone and with pain recognition training, improved all of the studied indicators, and a statistically significant difference between the groups was found in the assessment of fear of movement. 

### Strengths and Limitations

The strengths of this study were its randomization and adaptation to a clinical setting in the primary health care unit, which shows that it is possible to add pain neuroscience education to a daily clinical practice of physiotherapy. However, due to its explorative nature, the study cohort was too small and with relatively young and healthy participants, for example, those who did not need to use any prescribed or regular, unprescribed pain medication. The younger cohort in this study might also be a strength of this study, indicating that even relatively young people with NLBP benefit from the education in combination with the physiotherapy, particularly regarding the kinesiophobia aspects. The pilot approach that was taken is a limitation of this study. Another limitation is the absence of educational level, as this might have an impact on lower back pain *per se* and also on the possibility to understand the knowledge presented during the educational sessions. Furthermore, the combination of two different treatment approaches, due to their longer treatment duration, can generally lead to better results. Future studies should consider the addition of a “sham-PNE” intervention, which would complement the findings of the current study. Therefore, the results should be confirmed in larger and more heterogenous cohorts in regard to participants´ understanding of the PNE.

## 5. Conclusions

Physiotherapy, applied both with and without pain neuroscience education, improved the indicators of patients’ chronic low back pain, fear of movement and functional capacity in a cohort of primary health care patients with NLBP. Therefore, it can be concluded that the inclusion of psychoeducation in physiotherapy has a positive effect on the reduction of fear of movement. As a consequence, this might also improve other functions, such as self-care. Therefore, we suggest including pain recognition education in treatment plans for non-specific low back pain. We suggest an individualization of the prepared educative material, taking into account the differences in the patients’ perception of pain.

## Figures and Tables

**Table 1:** Organization of study.

**Participants with Low Back Pain (n = 30)**	
I group (n = 15)	II group (n = 15)
**I assessment**	
1. Assessment of pain intensity using the numeric rating scale (NRS); 2. Assessment of fear of movement using the Tampa scale; 3. Assessment of the impact of back pain on the patient’s functional capacity according to the Oswestry disability index; 4. Assessment of functional disability using the Roland–Morris questionnaire.	
**Intervention**	
I group (n = 15)	II group (n = 15)
1. Physiotherapy	**1. Physiotherapy**
(a) Spinal stabilization exercises; (b) Muscle strength training exercises; (c) Stretching exercises. Session duration—45 min/2 times per week for 10 weeks.	
	**2. The educational program about the pain and its recognition (2 times, each 30 min).**
**II assessment**	
**Analysis of results**	

**Table 2:** Methods used in the study.

Method	Description
**Numeric rating scale (NRS)**	This is used for the quantitative assessment of pain. The intensity of pain is compared before and after the intervention. At the same time, the effectiveness of the treatment for the patients is evaluated. The numeric rating scale (NRS) consists of 11 numbers on a scale from 0 to 10 [21]. Pain scoring: 0 points—no pain; 1–3 points—mild pain; 4–5 points—moderate pain; 6–8 points—severe pain; 9–10 points—very severe pain.
**Tampa scale of kinesiophobia**	The scale is used to assess fear of movement or re-injury in individuals who experience pain. Higher fear of movement scores are associated with depression and anxiety.The questionnaire consists of 17 questions, which have 4 answer options: Completely disagree (1 point); Disagree (2 points); I agree (3 points); I completely agree (4 points). The minimum score is 17, the maximum score is 68. The more points, the greater the fear of movement [22].
**Oswestry disability index**	Intended to assess the impact of back pain on the patient’s functional ability, including various life situations and daily activities. The questionnaire consists of 10 questions with 6 answers each (A-F). The letter of each answer is scored: A—0; B—1; C—2; D–3; E—4; F—5. Zero points is the highest rating for the feature, 50 points is the lowest. The obtained scores are multiplied by two and converted into percentages [23]. Assessment was adapted according to Samėnienė et al. [24]: 0–20 percent—minimal dysfunction; 2–40 percent—moderate dysfunction; 40–60 percent—severe dysfunction; 60–80 percent—disability; 80–100 percent—bed rest (or simulated symptoms).
**Roland** **–** **Morris disability questionnaire**	Intended to assess the impact of lower back pain on the patient’s functional condition. Based on the SIP (sickness impact profile) questionnaire. The effectiveness of the intervention is evaluated after the repeated filling of this questionnaire after the applied effect so as to monitor the patient’s functional status. Rating scale—0–24. The lower the score, the lower the impact of lower back pain on the functional state of the person [25].

**Table 3:** Demographic characteristics of the subjects and factors expressed in percentages (n), except for age which is presented in mean ± SD.

Demographic Factors		I Group (Physiotherapy)	II Group (Physiotherapy with PNE)	In General	Comparison between the Groups
Age		33.13 ± 4.37	33.8 ± 4.51	33.47 ± 4.38	U = 102.0; *p* = 0.683
Gender	Women	53.3 (8)	73.3 (11)	63.3 (19)	χ2(1) = 1.292; *p* = 0.256
	Men	46.7 (7)	26.7 (4)	36.7 (11)	
Do you often complain of pain in the lower back?	Every day	53.3 (8)	33.3 (5)	43.3 (13)	U = 86.0; *p* = 0.301
	Once or several times a week	33.3 (5)	40 (6)	36.7 (11)	
	More rarely	13.3 (2)	26.7 (4)	20 (6)	
Have you ever had a spinal injury or disease?	Yes	13.3 (2)	6.7 (1)	10 (3)	U = 105.0; *p* = 1.0
	No	86.7 (13)	93.3 (14)	90 (27)	
Do you have any co-morbidities?	Yes	13.3 (2)	-	6.7 (2)	U = 97.5; *p* = 0.483
	No	86.7 (13)	100 (15)	93.3 (28)	
Does the pain limit daily activities and functions?	Yes	100 (15)	93.3 (14)	96.7 (29)	U = 105.0; *p* = 1.0
	No	-	6.7 (1)	3.3 (1)	
Do you take medication for lower back pain?	Yes	26.7 (4)	20 (3)	23.3 (7)	U = 105.0; *p* = 1.0
	No	73.3 (11)	80 (12)	76.7 (23)	

## Data Availability

The data that support the findings of this study are available from the first author (E.L.) upon reasonable request.

## Supplementary Materials

The following supporting information can be downloaded at: https://www.mdpi.com/article/10.3390/jcm13072081/s1, Supplemental Figure S1: Flow diagram.

## References

[1] Hartvigsen J., Hancock M.J., Kongsted A., Louw Q., Ferreira M.L., Genevay S., Hoy D., Karppinen J., Pransky G., Sieper J. (2018). What low back pain is and why we need to pay attention. Lancet.

[2] Wu A., March L., Zheng X., Huang J., Wang X., Zhao J., Blyth F.M., Smith E., Buchbinder R., Hoy D. (2020). Global low back pain prevalence and years lived with disability from 1990 to 2017: Estimates from the Global Burden of Disease Study 2017. Ann. Transl. Med..

[3] Lietuvos Statistikos Departamentas (2018). Rodiklių Duomenų bazė—Oficialiosios Statistikos Portalas. https://osp.stat.gov.lt/statistikos-leidiniu-katalogas?publication=33980.

[4] O’Sullivan P.B., Caneiro J.P., O’Keeffe M., Smith A., Dankaerts W., Fersum K., O’Sullivan K. (2018). Cognitive Functional Therapy: An Integrated Behavioral Approach for the Targeted Management of Disabling Low Back Pain. Phys. Ther..

[5] Bardin L.D., King P., Maher C.G. (2017). Diagnostic triage for low back pain: A practical approach for primary care. Med. J. Aust..

[6] Sahin N., Karahan A.Y., Albayrak I. (2018). Effectiveness of physical therapy and exercise on pain and functional status in patients with chronic low back pain: A randomized-controlled trial. Turk. J. Phys. Med. Rehabil..

[7] Frizziero A., Pellizzon G., Vittadini F., Bigliardi D., Costantino C. (2021). Efficacy of Core Stability in Non-Specific Chronic Low Back Pain. J. Funct. Morphol. Kinesiol..

[8] Sipaviciene S., Kliziene I. (2020). Effect of different exercise programs on non-specific chronic low back pain and disability in people who perform sedentary work. Clin. Biomech..

[9] Smrcina Z., Woelfel S., Burcal C. (2022). A Systematic Review of the Effectiveness of Core Stability Exercises in Patients with Non-Specific Low Back Pain. Int. J. Sports Phys. Ther..

[10] Fatemi R., Javid M., Najafabadi E.M. (2015). Effects of William training on lumbosacral muscles function, lumbar curve and pain. J. Back Musculoskelet. Rehabil..

[11] Szulc P., Wendt M., Waszak M., Tomczak M., Cieslik K., Trzaska T. (2015). Impact of McKenzie Method Therapy Enriched by Muscular Energy Techniques on Subjective and Objective Parameters Related to Spine Function in Patients with Chronic Low Back Pain. Med. Sci. Monit..

[12] Shariat A., Cleland J.A., Danaee M., Kargarfard M., Sangelaji B., Tamrin S.B.M. (2018). Effects of stretching exercise training and ergonomic modifications on musculoskeletal discomforts of office workers: A randomized controlled trial. Braz. J. Phys. Ther..

[13] Shiri R., Coggon D., Falah-Hassani K. (2018). Exercise for the Prevention of Low Back Pain: Systematic Review and Meta-Analysis of Controlled Trials. Am. J. Epidemiol..

[14] Gordon R., Bloxham S. (2016). A Systematic Review of the Effects of Exercise and Physical Activity on Non-Specific Chronic Low Back Pain. Healthcare.

[15] Wood L., Hendrick P.A. (2019). A systematic review and meta-analysis of pain neuroscience education for chronic low back pain: Short-and long-term outcomes of pain and disability. Eur. J. Pain.

[16] Louw A., Zimney K., O’Hotto C., Hilton S. (2016). The clinical application of teaching people about pain. Physiother. Theory Pract..

[17] Puentedura E.J., Flynn T. (2016). Combining manual therapy with pain neuroscience education in the treatment of chronic low back pain: A narrative review of the literature. Physiother. Theory Pract..

[18] Robins H., Perron V., Heathcote L.C., Simons L.E. (2016). Pain Neuroscience Education: State of the Art and Application in Pediatrics. Children.

[19] Wijma A.J., Speksnijder C.M., Crom-Ottens A.F., Knulst-Verlaan J.M.C., Keizer D., Nijs J., van Wilgen C.P. (2018). What is important in transdisciplinary pain neuroscience education? A qualitative study. Disabil. Rehabil..

[20] Meise R., Ferreira Carvalho G., Thiel C., Luedtke K. (2023). Additional effects of pain neuroscience education combined with physiotherapy on the headache frequency of adult patients with migraine: A randomized controlled trial. Cephalalgia.

[21] Williamson A., Hoggart B. (2005). Pain: A review of three commonly used pain rating scales. J. Clin. Nurs..

[22] French D.J., France C.R., Vigneau F., French J.A., Evans R.T. (2007). Fear of movement/(re)injury in chronic pain: A psychometric assessment of the original English version of the Tampa scale for kinesiophobia (TSK). Pain.

[23] Fairbank J.C., Pynsent P.B. (2000). The Oswestry Disability Index. Spine.

[24] Samėnienė J M.T., Medzevičiūtė R., Valančiūtė A., Brazauskaitė L., Narauskas R. (2005). Nugaros skausmo įtaka paciento funkcinei būklei ir gyvenimo kokybei bei jo vertinimas reabilitacijoje. Skausmo Med..

[25] Roland M., Fairbank J. (2000). The Roland-Morris Disability Questionnaire and the Oswestry Disability Questionnaire. Spine.

[26] Iversen V.M., Vasseljen O., Mork P.J., Berthelsen I.R., Borke J.B., Berheussen G.F., Tveter A.T., Salvesen O., Fimland M.S. (2017). Resistance training in addition to multidisciplinary rehabilitation for patients with chronic pain in the low back: Study protocol. Contemp. Clin. Trials Commun..

[27] Moon H.J., Choi K.H., Kim D.H., Kim H.J., Cho Y.K., Lee K.H., Kim J.H., Choi Y.J. (2013). Effect of lumbar stabilization and dynamic lumbar strengthening exercises in patients with chronic low back pain. Ann. Rehabilitation Med..

[28] Boucher J.A., Preuss R., Henry S.M., Dumas J.P., Lariviere C. (2016). The effects of an 8-week stabilization exercise program on lumbar movement sense in patients with low back pain. BMC Musculoskelet. Disord..

[29] Gorji S.M., Mohammadi Nia Samakosh H., Watt P., Henrique Marchetti P., Oliveira R. (2022). Pain Neuroscience Education and Motor Control Exercises versus Core Stability Exercises on Pain, Disability, and Balance in Women with Chronic Low Back Pain. Int. J. Environ. Res. Public Health.

[30] Malfliet A., Kregel J., Coppieters I., De Pauw R., Meeus M., Roussel N., Cagnie B., Danneels L., Nijs J. (2018). Effect of Pain Neuroscience Education Combined With Cognition-Targeted Motor Control Training on Chronic Spinal Pain: A Randomized Clinical Trial. JAMA Neurol..

[31] Kim K.S., An J., Kim J.O., Lee M.Y., Lee B.H. (2022). Effects of Pain Neuroscience Education Combined with Lumbar Stabilization Exercise on Strength and Pain in Patients with Chronic Low Back Pain: Randomized Controlled Trial. J. Pers. Med..

[32] Watson J.A., Ryan C.G., Cooper L., Ellington D., Whittle R., Lavender M., Dixon J., Atkinson G., Cooper K., Martin D.J. (2019). Pain Neuroscience Education for Adults with Chronic Musculoskeletal Pain: A Mixed-Methods Systematic Review and Meta-Analysis. J. Pain.

[33] Wijma A.J., van Wilgen C.P., Meeus M., Nijs J. (2016). Clinical biopsychosocial physiotherapy assessment of patients with chronic pain: The first step in pain neuroscience education. Physiother. Theory Pract..

[34] Gul H., Erel S., Toraman N.F. (2021). Physiotherapy combined with therapeutic neuroscience education versus physiotherapy alone for patients with chronic low back pain: A pilot, randomized-controlled trial. Turk. J. Phys. Med. Rehabil..

[35] Siddall B., Ram A., Jones M.D., Booth J., Perriman D., Summers S.J. (2022). Short-term impact of combining pain neuroscience education with exercise for chronic musculoskeletal pain: A systematic review and meta-analysis. Pain.

