# Supplementary figures and images for: Effects of Pain Neuroscience Education and Physiotherapy on Chronic Low Back Pain, Fear of Movement and Functional Status: A Randomised Pilot Study
Source: J Clin Med. 2024 Apr 3;13(7):2081. doi: 10.3390/jcm13072081 (PMC11012892; doi:10.3390/jcm13072081)

## CONSORT 2010 Flow Diagram

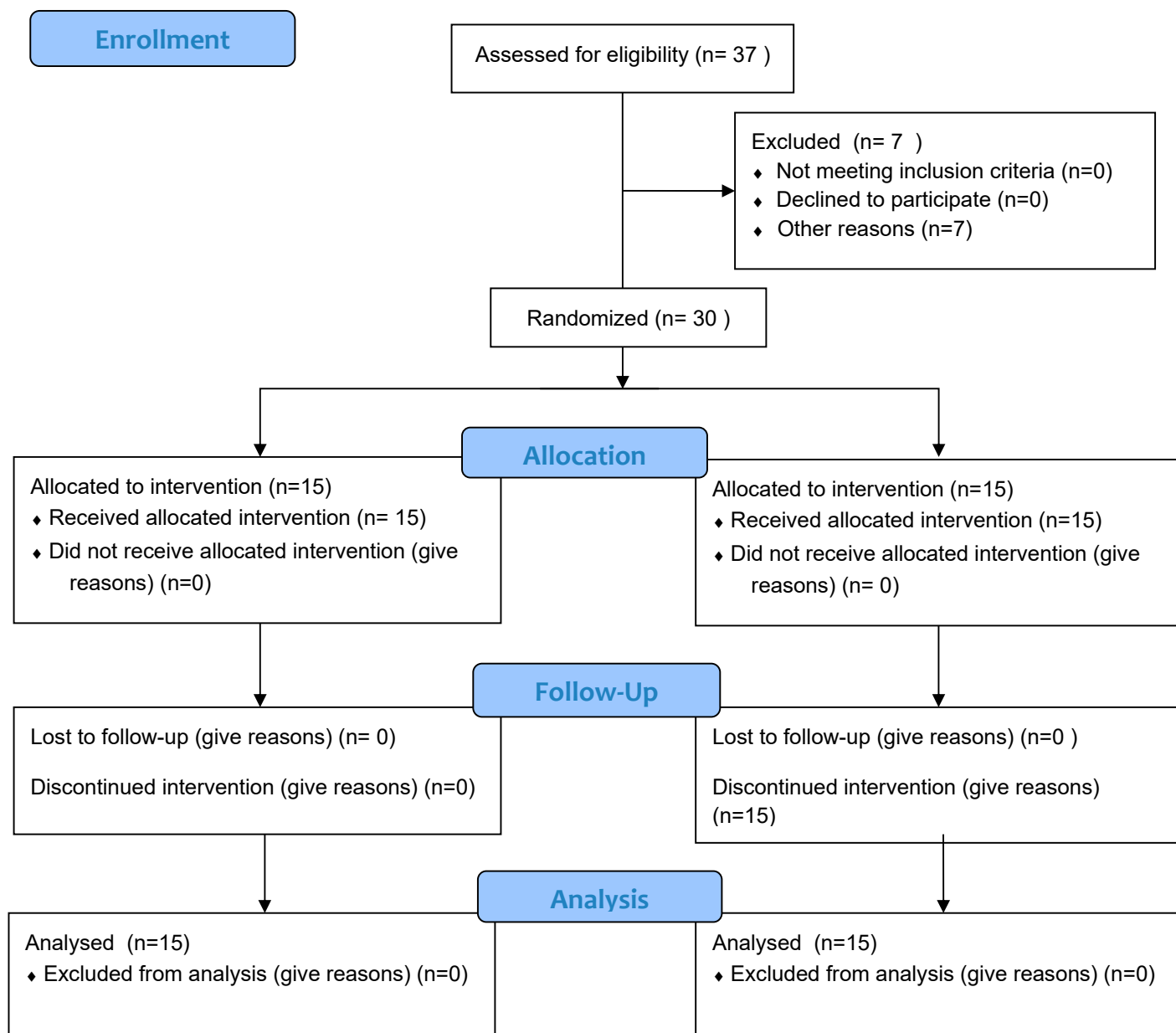

Supplement: Supplementary file 1 [file jcm-13-02081-s001.zip › jcm-2827125-supplementary.pdf]
